# Trans-arterial embolisation (TAE) in haemorrhagic pelvic injury: review of management and mid-term outcome of a major trauma centre

**DOI:** 10.1186/s42155-018-0031-3

**Published:** 2018-11-24

**Authors:** Amir Awwad, Permesh Singh Dhillon, Greg Ramjas, Said B. Habib, Waleed Al-Obaydi

**Affiliations:** 1 Interventional Radiology, Queen’s Medical Centre, Nottingham University Hospitals NHS Trust, Nottingham, NG7 2UH UK; 2 NIHR Nottingham Biomedical Research Centre, Sir Peter Mansfield Imaging Centre, School of Medicine, University of Nottingham, Nottingham, NG72UH UK; 3 Radiology Department, Royal Papworth Hospital NHS Foundation Trust, Cambridge, CB23 3RE UK; 4 Interventional Radiology, Royal Derby Teaching Hospitals NHS Foundation Trust, Uttoxeter Road, Derby, DE22 3NE UK

**Keywords:** Trans-arterial embolisation, Intervention, Pelvic trauma, Haemorrhage, Gelfoam, Coils

## Abstract

**Background:**

Management of pelvic fracture associated haemorrhage is often complex with high morbidity and mortality rates. Different treatment options are used to control bleeding with an on-going discussion in the trauma community regarding the best management algorithm.

**Main body:**

Recent studies have shown trans-arterial embolisation (TAE) to be a safe and effective technique to control pelvic fracture associated haemorrhage. Computed tomography (CT) evidence of active bleeding, haemodynamic instability, and pelvic fracture patterns are amongst important indicators for TAE.

**Conclusion:**

Herein, we aim to provide a comprehensive literature review of the effectiveness of TAE in controlling haemorrhage secondary to pelvic fracture according to the indications, technique and embolic agents, and outcomes, whilst incorporating our Level 1 major trauma centre’s (MTC) results between 2014-2017.

**Electronic supplementary material:**

The online version of this article (10.1186/s42155-018-0031-3) contains supplementary material, which is available to authorized users.

## Background

Trauma is a leading cause of death in the young population (Sauaia et al., 1995). Pelvic fractures are present in up to 9.3% of patients with high energy blunt trauma, especially following road traffic accidents (Demetriades et al., 2002; El-Haj et al., 2013; Katsura et al., 2013; Hauschild et al., 2012). Pelvic fractures are often associated with other visceral and vascular injuries (Ertel et al., 2001). Bleeding is a common occurrence in patients with pelvic ring fracture and it is the second most common cause of death after brain injury in these patients (Sauaia et al., 1995; Kauvar et al., 2006). Although the mortality rate in patients with pelvic fractures can be as high as 13.5%, it significantly rises to 40–60% in patients with pelvic fracture and haemodynamic instability secondary to haemorrhage (Demetriades et al., 2002; Starr et al., 2002).

It is imperative during the initial management of polytrauma cases to lower the mortality rate secondary to bleeding. One of the main causes of early mortality is massive haemorrhage and shock, while later mortality is predominantly due to adult respiratory distress syndrome and multi organ failure, the latter as a result of massive blood transfusion with the subsequent inflammatory response (Sauaia et al., 1995; Fangio et al., 2005; Smith et al., 2007; Wong et al., 2000). Therefore timing is crucial in the management of these patients as early identification and control of pelvic haemorrhage might result in the reduction of pelvic fracture-related mortality and improve outcome (Agolini et al., 1997).

Previously published studies compared different methods of intervention including open laparotomy, pelvic packing, pelvic binders and trans-arterial embolisation (TAE), however such comparison proved difficult for several reasons (Burlew et al., 2011). This mainly is due to the variation in the availability of embolisation services between different hospitals, management algorithms between different hospitals and inconsistent criteria for patients’ referral for embolisation. Whilst there is growing evidence that suggests TAE for the treatment of acute haemorrhage in trauma patients is a safe and cost-effective method, current national guidelines identified the lack of high-level evidence with regards to the effectiveness of TAE. This is likely due to the non-existence of prospective and/or randomized studies or trials given the challenging ethical issues associated with this type of emergency research (NICE, 2016). Therefore, we set out to provide a comprehensive literature review of the effectiveness of TAE in controlling haemorrhage secondary to pelvic fracture, whilst incorporating our Level 1 major trauma centre’s (MTC) results with mid-term outcome analysis.

## Indications

TAE has been more frequently used to control pelvic fracture related bleeding since it was described in the literature in the 1970s with a high success rate of 80–100% (Fangio et al., 2005; Agolini et al., 1997; White et al., 2009; Velmahos et al., 2000a), especially where mechanical compression by pelvic packing and pelvic binders are ineffective (Fangio et al., 2005; Agolini et al., 1997; White et al., 2009; Velmahos et al., 2000a; Velmahos et al., 2002). It is shown to be effective against arterial haemorrhage, which is the source of bleeding in about 15% of pelvic fracture associated haemorrhage (Eastridge et al., 2002). TAE is also equally effective in cases of pelvic re-bleeding following initial angiography +/− embolisation in the emergency setting (Cullinane et al., 2011).

**Fig. 1:**
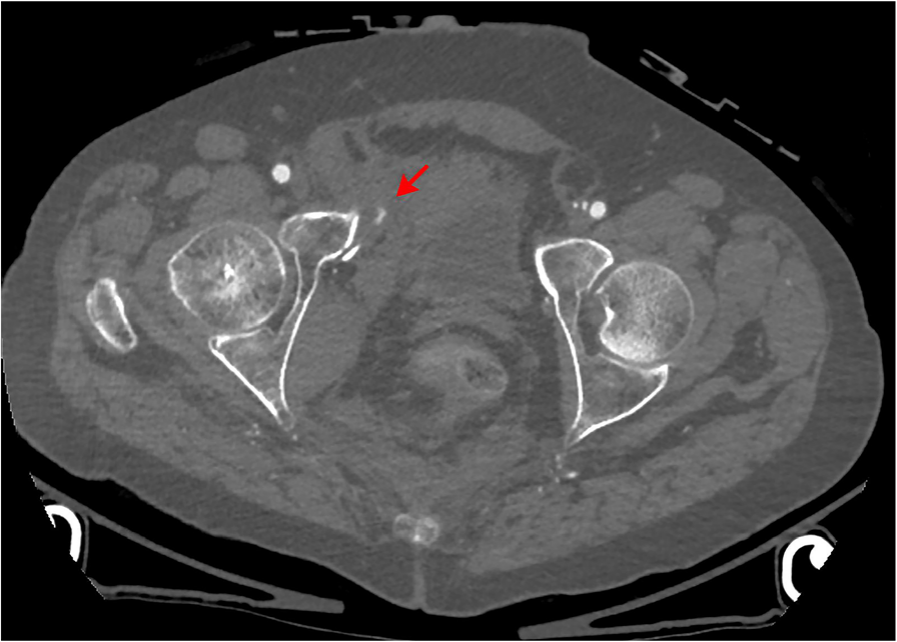
A 64-year-old polytrauma female patient admitted with reduced consciousness, pelvic fracture dislocation and right pelvic haematoma. Urgent arterial phase trauma CT scan (1 mm slice thickness) axial image showing a high-density focus (red arrow) representing contrast extravasation in keeping with active bleeding, see online Additional file 1

**Fig. 2:**
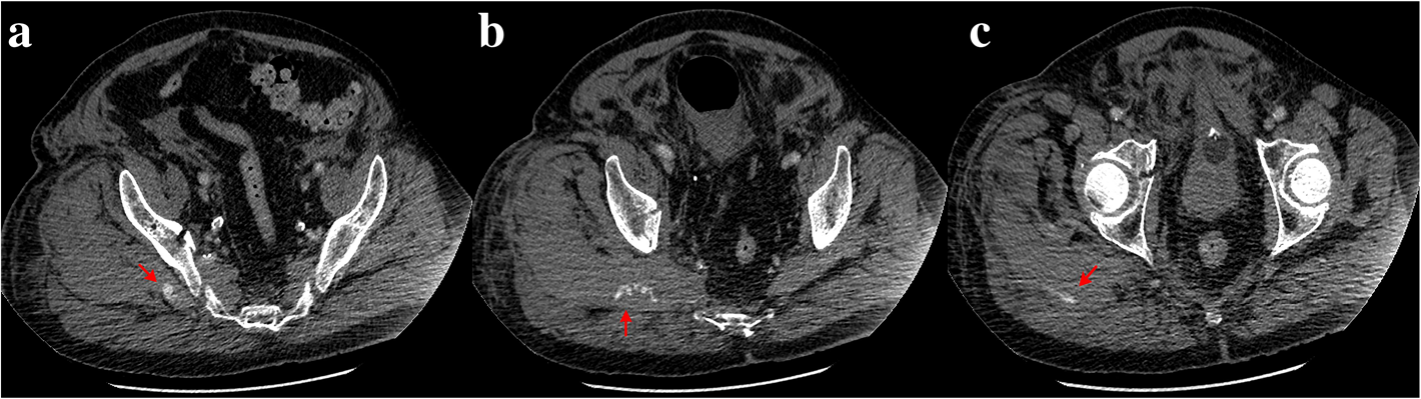
**a-c** An 83-year-old road traffic collision (RTC) male patient admitted with significant pelvic fracture and haemodynamic instability (on Warfarin). Series of axial images (**a**-**c**) from an urgent arterial phase CT scan (1 mm slice thickness) showing extensive right gluteus medius hematoma (14 × 4.5 cm) with high-density foci of contrast extravasation spreading (red arrows) inferolateral to an acutely fractured right iliac bone, see online Additional file 2

It has been reported that any contrast extravasation on a computed tomography (CT) scan is highly predictive of active bleeding with sensitivity and specificity values ranging between 66 and 90% and 85–98% respectively (Ierardi et al., 2015; Miller et al., 2003). Furthermore, the exact haemorrhagic site can often be identified on CT and correlates closely with the angiographic findings, which guides the time critical TAE treatment by the interventional radiologist. Previous studies have indicated the need for TAE based on any contrast extravasation seen on CT, however in another study nearly half of the patients with contrast extravasation on CT did not require embolisation (Stephen et al., 1999; Michailidou et al., 2012). In our study, only 11 patients had active contrast extravasation on CT, shown as a high density focus due to a leaking blush/jet of contrast (Figs. 1 and 2). However, a bleeding point was demonstrated in 19 patients on angiography (Table 2). The discrepancy between CT and angiographic findings in demonstrating active bleeding has been postulated to be due to vessel spasm, which is thought to be secondary to local inflammatory response generated by bleeding or hypotension (Dietrich & Dacey, 2000). Hence, it is imperative to consider proceeding to angiography +/− TAE following a trauma CT scan if the clinical suspicion remains high of underlying bleeding based on the type of pelvic fracture and haemodynamic stability.

There have been attempts to stratify groups of patients who would benefit from TAE in terms of their haemodynamic status, transfusion requirement, pelvic fracture pattern and other associated injuries (El-Haj et al., 2013; Abrassart et al., 2013; Fu et al., 2013; Barentsz et al., 2011; Metsemakers et al., 2013).

Previous studies have identified pelvic fracture instability and various fracture patterns (Young-Burgess classification system) such as types 2 and 3 anterior-posterior, type 3 lateral and vertical compression pelvic injuries or combined mechanisms are strongly associated with arterial haemorrhage, and should be considered when deciding the primary method of haemorrhage control (Ierardi et al., 2015; Manson et al., 2010). On the contrary, few authors did not find any significant correlation between pelvic fracture severity or grading and the need for TAE or haemodynamic instability (Comai et al., 2016). These differences remain controversial and can be accounted for by the difference in management algorithms and sample sizes.

In the trauma setting, pelvic injuries often co-exist with other visceral injuries, which may impact the initial management and final outcome. The injury severity score (ISS) is an established scoring system used to quantify the severity of injuries for a patient. Each injury is given a score of between 1 (minor injury) and 6 (incompatible with life) and the total ranges from 1 to 75 (TARN, n.d.). Few studies have demonstrated an association between high ISS scores (> 25) and a favourable outcome following TAE (Karadimas et al., 2011). Some authors have argued that in certain cases where active bleeding is identified secondary to unstable pelvic fractures and concomitant intra-abdominal injuries, the treatment option would be simplified by surgical intervention (Demetriades et al., 2002). However, other studies suggest that exploratory laparotomy has limited success in identifying bleeding sites and ligating small affected arterial branches (Niola et al., 2012). TAE has also been shown to be equally effective with a lower mortality rate, to deal with concomitant pelvic and intra-abdominal bleeding (Fang et al., 2011). Hence, given its less invasive nature, many view TAE as the favoured treatment option.

Increased initial transfusion requirements of > 0.5unit/hour and haemodynamic instability, defined as a systolic blood pressure (SBP) below 90 mmHg, tachycardia and capillary refill time > 2 s have been shown to be further predictors for TAE (Karadimas et al., 2011). The recent Western Trauma Association update has outlined TAE as the primary method of haemorrhagic control in patients resistant to fluid resuscitation and mechanical stabilisation (Tran et al., 2016). However, due to inconsistencies in the availability and delays in performing TAE between institutions, the decision to take the patient to theatre for pre-peritoneal packing or angiography suite for TAE would be made following discussion between the trauma team members taking into consideration the CT findings and the haemodynamic status of the patient (Lustenberger et al., 2015). Interestingly, age (above 60) has also been documented as an independent predictor for TAE, regardless of the haemodynamic status (Kimbrell et al., 2004). In our study, there was no significant correlation between the haemodynamic stability, ISS and the embolisation or mortality outcome as compared to some studies (Katsura et al., 2013; Abrassart et al., 2013; Matityahu et al., 2013; Tanizaki et al., 2014) .

In select cases, re-bleeding may occur following initial therapy, with a reported rate of up to 9.7% (Ierardi et al., 2016; Gourlay et al., 2005; Shapiro et al., 2005). Authors have identified specific risk factors to allow early identification of a re-bleed, which include pre-procedural haemoglobin of < 7.5 mg/dl, initial finding of greater than two bleeding sites, absence of concomitant intra-abdominal visceral injury, post-procedural transfusion requirement of > 6 units RBC and continued haemodynamic instability, super-selective embolisation and a persistent base deficit (Gourlay et al., 2005; Shapiro et al., 2005; Fang et al., 2009). In these cases, operators may opt for selective or non-selective embolisation according to preference.

## Management

**Fig. 3:**
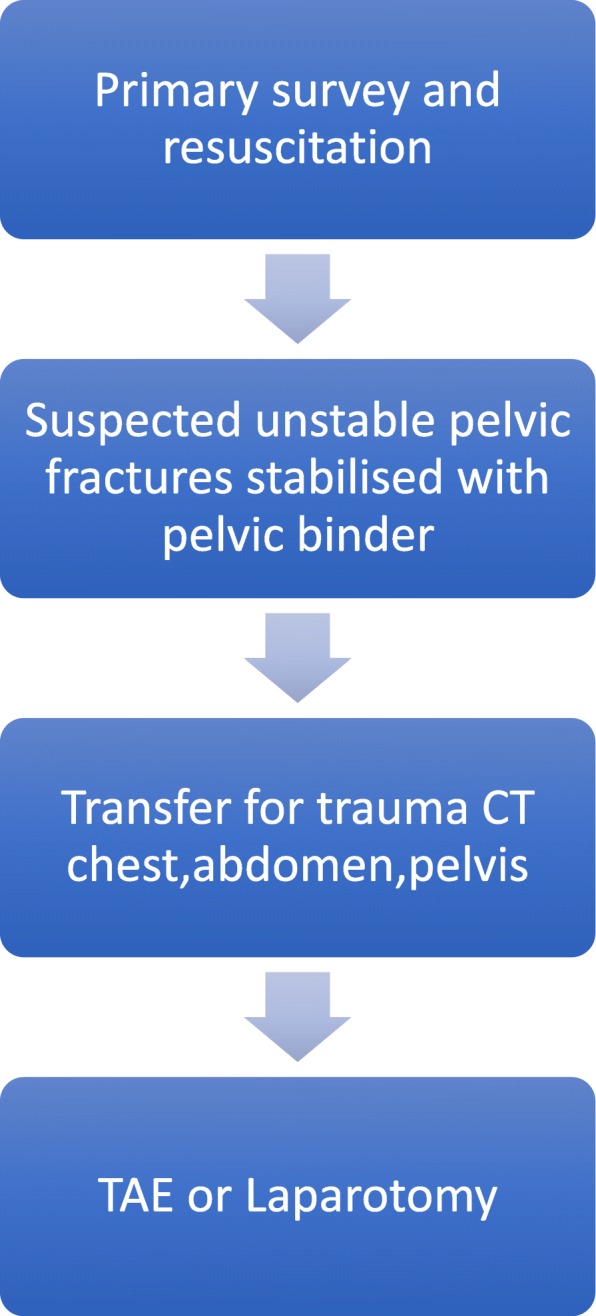
Flow chart representing our major trauma centre’s management algorithm for haemodynamically unstable patients with traumatic pelvic haemorrhage. Adapted from the 2012 CIRSE guidelines (Chakraverty et al., 2012). CT denotes computed tomography; TAE denotes trans-arterial embolisation

There are multiple proposed management algorithms for pelvic trauma patients, which may vary between institutions. Our institution follows the trauma algorithm as demonstrated (Fig. 3) (Chakraverty et al., 2012).

### Imaging protocol

The trauma algorithm at our institution is also derived from NICE guidelines on pelvic imaging in setting of poly-trauma (suspected pelvic or acetabular fracture) transferred to an MTC. Our local approach recommends that the trauma patient undergoes a dual phase CE-CT scan as a first-line imaging in adults with suspected high-energy pelvic fractures. Our conventional trauma imaging protocol is performed withan intravenous injection of 90mls contrast medium, given at a rate of 3.5 ml/sec. Two spiral acquisitions CT chest/abdomen/pelvis are obtained, the first at 20s to obtain an arterial phase and followed by a porto-venous (PV) phase at 70s. Alternatively, some institutions have adopted the Camp Bastion protocol, which was initially practiced by the Armed Forces for trauma injuries at Camp Bastion, Afghanistan (Graham, 2012). This protocol constitutes a bi-phasic contrast enhanced whole body CT, where a single split-rate injection is administered at two different rates (1/3 at 1.5 ml/sec, 2/3 at 3.5mls) before scanning at 70s (Hakim et al., 2016). This has the advantage of reducing radiation and scan time. Recent studies have demonstrated comparable imaging quality using bi-phasic protocol with single CT acquisition (Hakim et al., 2016; Beenen et al., 2015).

At our trauma centre, an active bleeding site is identified as a high density focus due to a leaking blush/jet of contrast seen on the arterial phase scan, with further pooling of contrast on the PV phase. Other forms of arterial injury such as pseudo-aneurysm formation, arterio-venous fistula, dissection or transection have also been documented (Ptohis et al., 2017; Frandon et al., 2016). Secondary signs of bleeding include retroperitoneal hematoma, which may be present without signs of contrast extravasation.

### Angiography & Embolisation Technique

Regarding the angiography procedure itself, most authors described a standard approach via the common femoral artery (CFA). Under local anaesthesia in the groin, the CFA is punctured with a needle and a 4 or 5French sheath is then introduced using the Seldinger technique to secure access into the arterial tree. Our local practice is to start with an aortogram to delineate the anatomy first, then guided by CT positive findings a direct catheterisation of the suspected internal iliac artery would follow. Contrast is then injected to identify the bleeding point, identified as a focus of contrast extravasation/blush. Digital subtraction angiography (DSA) is used to guide the operator along the arterial map (49). Standard catheter use is by default recommended at our institution. However, if the bleeding point is not evident, further selective micro-catheterisation and angiogram of the pelvic arterial branches on the affected side is necessary to identify the bleeding point. Once this is evident, the bleeding artery is embolised accordingly using a variety of embolic material (e.g. micro-coils and/or glue), which is further discussed below.

Interrogating the internal iliac artery (IIA) branches is crucial as they are a common source of bleeding (Fangio et al., 2005; Smith et al., 2007). Bleeding from branches of the external iliac and common femoral arteries has also been described in previous studies (Barentsz et al., 2011; Metsemakers et al., 2013). Therefore, exploring these arteries is essential to avoid missing a bleeding source. Occasionally, when there are multiple distal bleeding sites identified, the operator may opt to perform proximal or non-selective embolisation using temporary embolic materials to save valuable time in haemodynamically unstable patients.

**Fig. 4:**
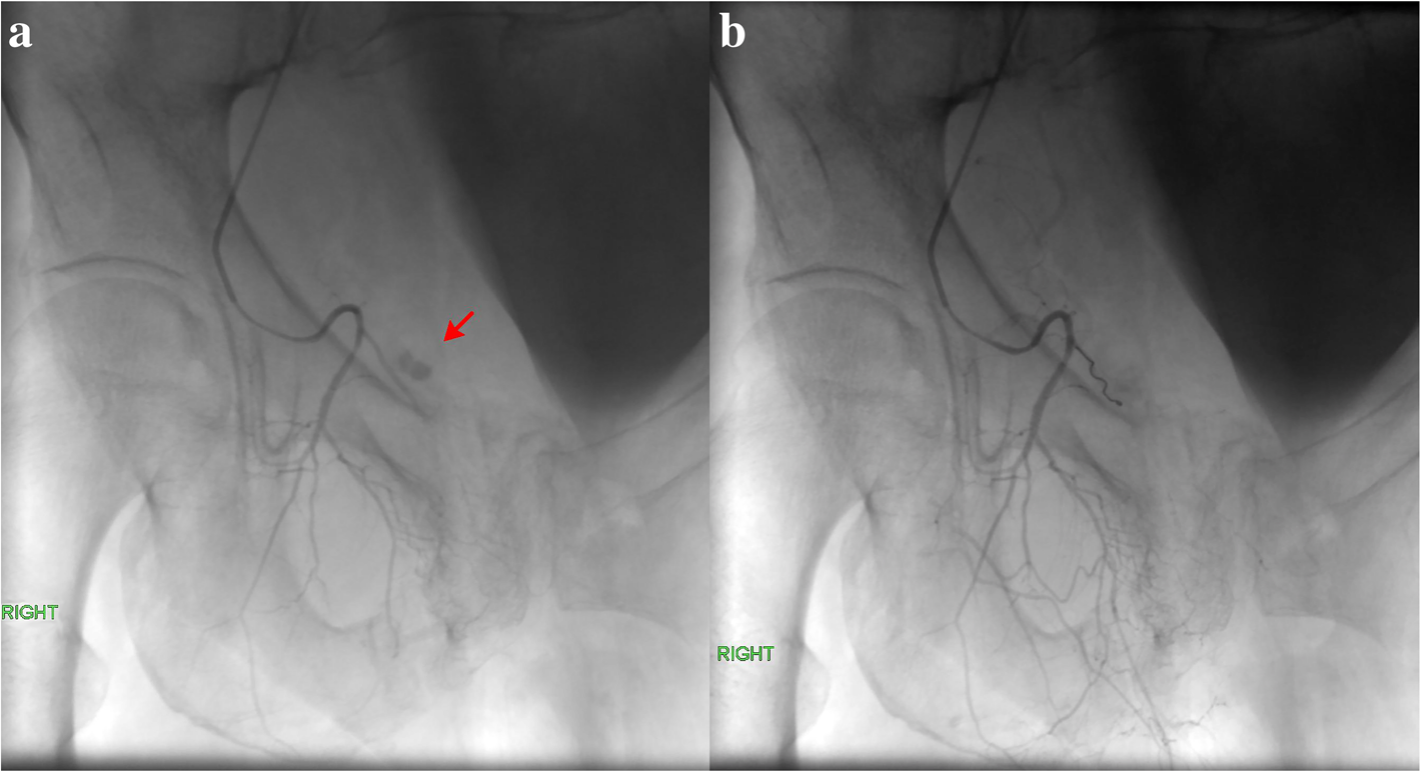
**a**, **b** A 64-year-old polytrauma female patient admitted with reduced consciousness, pelvic fracture dislocation and right pelvic hematoma. Pre-embolisation angiography image (**a**) showing active bleeding from a medial branch of right inferior epigastric artery (red arrow). Post selective embolisation (Gelfoam and single microcoil) angiographic image (**b**) showing cessation of bleeding with complete haemodynamic stability, see online Additional file 3

**Fig. 5:**
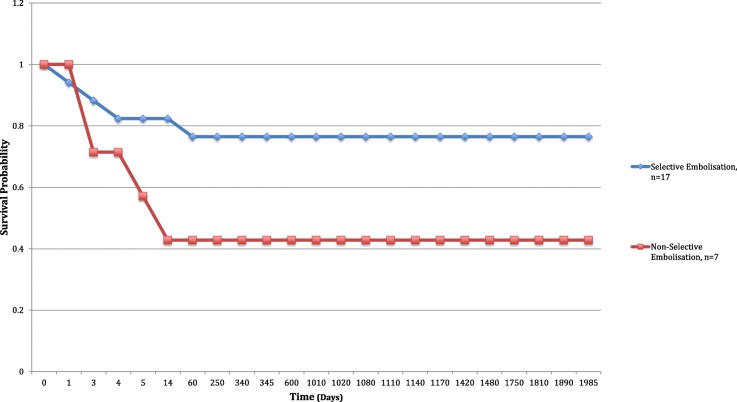
Survival analysis of patients with pelvic trauma who underwent selective and non-selective TAE

In our study, TAE was the primary management line to control bleeding in 22 patients while 2 patients initially underwent laparotomy followed by TAE. Apart from one patient with a single actively bleeding branch of the external iliac artery, various branches of the internal iliac artery were the main culprits in the remaining cohort of patients. In every TAE, the operator confirmed a well-sealed arterial defect and stoppage of contrast leakage on a final DSA run. Selective embolisation performed in 17 of 24 cases (Fig. 4) was associated with improved survival outcome (Fig. 5).

### Embolic agents

**Fig. 6:**
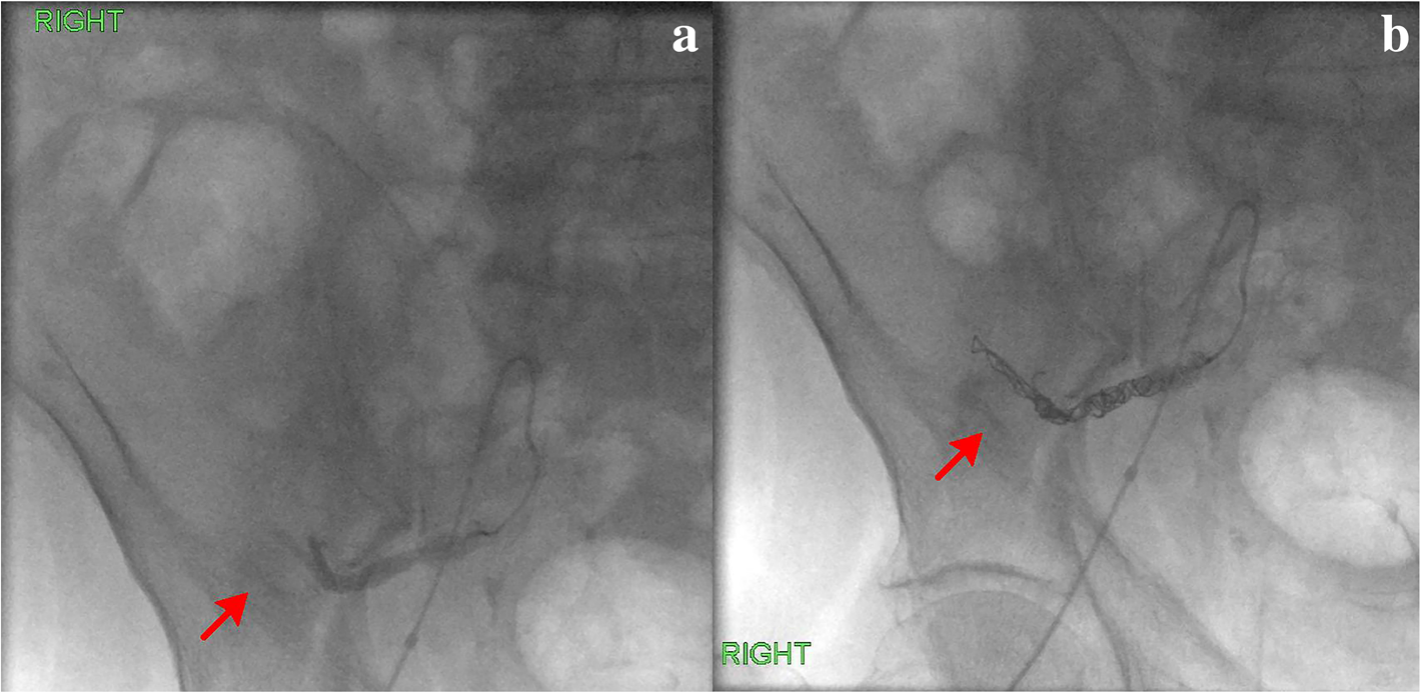
**a**, **b** An 83-year-old road traffic collision (RTC) male patient admitted with significant pelvic fracture and haemodynamic instability (on Warfarin). Pre-embolisation angiography image (**a**) showing active intra-muscular contrast extravasation from posterior trunk of right internal iliac artery. Gelfoam and several microcoils were deployed (red arrows) to seal the posterior trunk and stabilize patient’s haemodynamics as shown in post-embolisation image (**b**), see online Additional files 4 and 5

**Fig. 7:**
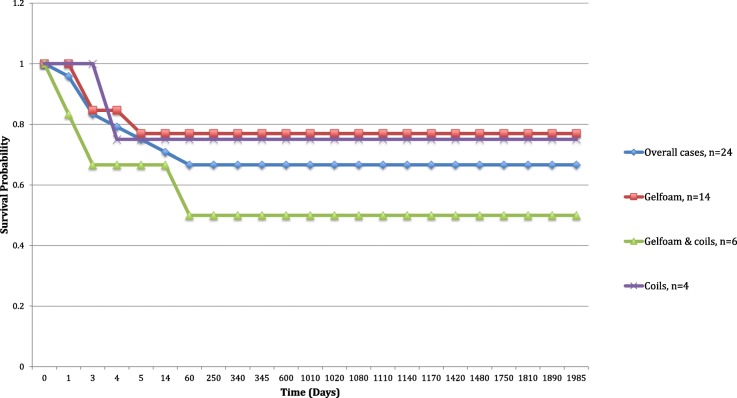
Survival analysis of patients with pelvic trauma who underwent TAE including subgroups of materials used (Gelfoam, Gelfoam and Coils, Coils)

Various embolic agents are available at the operator’s disposal. Gelfoam and coils seem to be the most commonly used materials, either as a single agent or a combination of the two, as was the case in this study (Table 3, Figs. 4, 6, 7). Gelfoam is a biodegradable gelatine sponge, which can be cut to size, and is mixed with contrast and normal saline prior to delivery. Gelfoam remains the most popular choice as it is a temporary embolic agent, which lasts for 7–21 days, and is relatively easy and economical to use (Frevert et al., 2008; Suzuki et al., 2005). A previous study also showed no long or short-term complications associated with the use of Gelfoam (Travis et al., 2008).

Coils and vascular Amplatzer plugs (self-expandable nitinol occlusion device) are frequently used for more definitive and targeted embolisation of distal vessels. Metallic coils come in various sizes and are generally coated with thrombogenic material (fibrogenic fibers) or are uncovered. They allow rapid mechanical occlusion of the vessel as they are injected through the microcatheter. Several coils are usually required to conform into an occlusive coil ball, which creates a scaffold for thrombosis (Frandon et al., 2016). However, extensive coil usage precludes distal vessel access in cases of re-bleeding.

Liquid agents such as Glue (n-butylcyanoacrylate) or Onyx may be considered for very distal vessels and can be used in cases of re-bleeding. However, due to increased costs, difficulties in controlling the amount used, slow administration and reflux of the agent upstream, the role of liquid agents in a time critical procedure is limited (Frandon et al., 2016).

## Outcome

### Our local experience

Between January 2014 to June 2017, A total of 24 adult patients [17 male, 7 female, mean age 55 (range 19–98 years)] underwent TAE to treat pelvic fracture associated haemorrhage. The mechanism of injury was road traffic accident in 18 patients; the other 6 were fall from height. Upon arrival to the emergency department, first-line trauma CT is performed as per local protocol, *n* = 23 received a local trauma CT and one case underwent a trauma CT elsewhere before transfer to our MTC (images were accessible locally) The decision to refer for TAE is not only based on CT finding of an active arterial pelvic bleed. It is also judged by other parameters such as ongoing clinical deterioration, severity of trauma and haemodynamic instability, all suggestive of continuous bleeding. Almost 58% of patients were haemodynamically unstable with manifestations of acute haemorrhagic shock. Isolated pelvic fracture was present in 5 patients; while the majority of patients had multiple associated injuries [head (*n* = 8), spinal (*n* = 9), chest (*n* = 15), and abdominal injuries (*n* = 5)]. The average ISS was 47 (range 25–60). Primary outcome measure was bleeding cessation at the end of procedure (100%) and secondary outcomes included post procedure early, late complications (0%) and mortality (29%).

Tables 1, 2, 3 summarize the patients’ clinical characteristics, computed tomography (CT) and angiographic findings and type of embolisation performed.

**Table 1:** Clinical characteristics of patients with pelvic trauma who underwent embolisation

Characteristics		Number, *n* (%)
Gender	Male	17 (71%)
	Female	7 (29%)
Age, median (yrs)		55
Injury severity score, median		47
Haemodynamics	Stable	10 (42%)
	Unstable	14 (58%)
Local Trauma CT	Yes	23 (96%)
	No	1 (4%)
Time, median (mins)	Admission to Embolisation	199
Mortality within 30 days	Yes	7 (29%)
	No	17 (71%)
Complications	Yes	0
	No	24 (100%)

**Table 2:** Comparison between CT and Digital Subtraction Angiography (DSA) findings with or without contrast extravasation in pelvic trauma patients who underwent embolisation

	CT + ve	CT –ve	Total	Diagnostic Statistical Evaluation of Trauma CT
DSA + ve	10	9	19	Sensitivity = 90.9% (95% CI = 58.7–99.8%)
DSA –ve	1	4	5	Specificity = 30.8% (95% CI = 9.1–61.4%)
Total	11	13	24	PLR = 1.3 (95% CI = 0.9–2%)
				NLR = 0.3 (95% CI = 0.04–2.3%)
				PPV = 52.6% (95% CI = 42.5–62.6%)
				NPV = 80% (95% CI = 34.2–96.9%)

*CI* confidence interval, *PLR* positive likelihood ratio, *NLR* negative likelihood ratio, *PPV* positive predictive value, *NPV* negative predictive value

**Table 3:** Types of material used for selective and non-selective embolisation in pelvic trauma patients

Embolic Agent	Level of Embolisation		Total
	Selective Embolisation	Non-selective Embolisation	
Gelfoam	8	6	14
Gelfoam and Coils	5	1	6
Coils	4	0	4
Total	17	7	24

### Effectiveness of TAE

Different measures have been described to define embolisation success. Some studies have reported procedural success as lack of subsequent intervention, reduction in blood transfusion requirement and/or reduction in mortality. In other studies, TAE success was confirmed with the cessation of bleeding at the end of procedure (Katsura et al., 2013; Hauschild et al., 2012; Barentsz et al., 2011; Metsemakers et al., 2013). TAE has been shown to be 85–97% effective in controlling haemorrhage secondary to pelvic trauma (Cullinane et al., 2011). In our retrospective review, we used the lack of contrast extravasation at the end of the procedure, and bleeding cessation was achieved in all of the cases, giving an overall success rate of 100%.

Treatment success in the form of non-requirement for subsequent intervention has reported rates of up to 95% (Velmahos et al., 2002; Ierardi et al., 2015). This is due to previous studies suggesting secondary bleeding following initial embolisation may be due to cessation of vessel spasm. Vessel spasm is thought to be secondary to local inflammatory response generated by bleeding or hypotension (Dietrich & Dacey, 2000). Hence these results may not directly reflect the efficacy of TAE itself.

Additionally, the success of embolisation may be influenced by the coagulopathy present in 25–40% of polytrauma patients, which is directly related to the injury severity score, and is believed to be secondary to aggressive resuscitation with crystalloids and depletion of clotting factors (Brohi et al., 2003; Cosgriff et al., 1997). Using mortality or blood transfusion requirement as a marker of treatment success is controversial, as most patients have associated extra pelvic injuries, which can be the leading cause of death. The mortality rate of 29% at 30 days in this study was comparable to others which range from 17 to 47%, while the most common causes were adult respiratory distress syndrome and multi organ failure (Agolini et al., 1997; Ierardi et al., 2015; Niola et al., 2012).

It has been established that the time taken from admission to TAE influences success rates, with increasing delays worsening the morbidity and mortality outcomes (NICE, 2016). A previous study reported improved survival rate when TAE was performed within 1 h from admission (Agolini et al., 1997; Tanizaki et al., 2014). However, the availability of IR services varies between hospitals and may be further limited by out of hours access. Where services are available 24 h a day in MTCs, the recently National Institute of Clinical Excellence (NICE) guidance have identified areas of improvement with time to access that should be in line with that of surgery (NICE, 2016). Increasingly, the need for hybrid theatres has been advocated to reduce the delays and improve patient outcome (Tran et al., 2016).

We reported a median time from admission to embolisation of 199 mins (range 82–1285 min), which was comparable to previous studies (Schwartz et al., 2014). The time to embolisation was partly over-estimated and confounded by a couple of cases that were taken straight from door to theatre, prior to embolisation.

### Complications of TAE

Although endovascular embolisation is considered to be a safe technique, there are reports in the literature of complications (Velmahos et al., 2000a; Velmahos et al., 2000b). Puncture site complications due to poor closure may result in pseudo-aneurysm formation or groin hematoma (Frevert et al., 2008). Few studies have also reported post procedure complications such as gluteal, bladder, femoral head, and skin necrosis (Suzuki et al., 2005; Obaro & Sniderman, 1995; Sieber, 1994). Other complications include paresis, impotence and surgical wound compromise have been described after embolisation in the pelvic region (Travis et al., 2008; Lee et al., 2000). Unintended or non-target vessel embolisation could account for these findings.

Alternatively, the complications may be secondary to the type of embolisation performed; selective or non-selective. One report suggested higher complications rate with non-selective embolisation, however the difference did not reach statistical significance (Travis et al., 2008). Other studies suggested that bilateral non-selective embolisation of the internal iliac arteries is not associated with increased risk of complications (Fu et al., 2013; Auerbach et al., 2012). Due to the rich collateral arterial supply within the pelvis, proximal embolisation can be safely performed without causing major complications. In our study, a majority (71%) of cases underwent selective embolisation, with no complications reported in the entire study population and there was a greater survival probability amongst these patients. In general, embolisation will create ischemia and necrosis that should be limited to the least possible area. Therefore, selective embolisation of the bleeding point demonstrated at the time of angiography should be considered.

## Conclusion

TAE is a safe and effective technique in controlling haemorrhage associated with pelvic fracture. Hence, it should be considered once arterial bleeding is suspected based on clinical, biochemical and radiological findings.

## Additional files

Additional file 1: Additional cine files 1. (AVI 15004 kb)

Additional file 2: Additional cine files 2. (AVI 4977 kb)

Additional file 3: Additional cine files 3. (AVI 19747 kb)

Additional file 4: Additional cine files 4. (AVI 14834 kb)

Additional file 5: Additional cine files 5. (AVI 3527 kb)

## Abbreviations

CE-CT: Contrast-enhanced computed tomography
CFA: Common femoral artery
DSA: Digital subtraction angiography
IIA: Internal iliac artery
ISS: Injury severity score
MTC: Major trauma centre
NICE: National institute of clinical excellence
PV: Porto-venous
SBP: Systolic blood pressure
TAE: Trans-arterial embolisation

## References

[CR1] Abrassart S, Stern R, Peter R (2013). Unstable pelvic ring injury with hemodynamic instability: what seems the best procedure choice and sequence in the initial management?. Orthop Traumatol Surg Res.

[CR2] Agolini SF, Shah K, Jaffe J, Newcomb J, Rhodes M, Reed JF (1997). Arterial embolization is a rapid and effective technique for controlling pelvic fracture hemorrhage. J Trauma.

[CR3] Auerbach Andrew D., Rehman Saqib, Kleiner Matthew T. (2012). Selective Transcatheter Arterial Embolization of the Internal Iliac Artery Does Not Cause Gluteal Necrosis in Pelvic Trauma Patients. Journal of Orthopaedic Trauma.

[CR4] Barentsz MW, Vonken EPA, van Herwaarden JA, Leenen LPH, Mali WPTM, van den Bosch MAAJ (2011). Clinical outcome of intra-arterial embolization for treatment of patients with pelvic trauma. Radiol Res Pract.

[CR5] Beenen LFM, Sierink JC, Kolkman S, Nio CY, Saltzherr TP, Dijkgraaf MGW et al (2015) Split bolus technique in polytrauma: a prospective study on scan protocols for trauma analysis. Acta Radiol 10.1177/0284185114539319 25033993

[CR6] Brohi K, Jasmin S, Heron M, Coats T (2003). Acute traumatic coagulopathy. J Trauma.

[CR7] Burlew CC, Moore EE, Smith WR, Johnson JL, Biffl WL, Barnett CC (2011). Preperitoneal pelvic packing/external fixation with secondary angioembolization: optimal care for life-threatening hemorrhage from unstable pelvic fractures. J Am Coll Surg.

[CR8] Chakraverty S, Flood K, Kessel D, McPherson S, Nicholson T, Ray CE (2012). CIRSE guidelines: quality improvement guidelines for endovascular treatment of traumatic hemorrhage. Cardiovasc Intervent Radiol.

[CR9] Comai A, Zatelli M, Haglmuller T, Bonatti G (2016). The role of Transcatheter arterial embolization in traumatic pelvic hemorrhage: not only pelvic fracture. Cureus.

[CR10] Cosgriff Ned, Moore Ernest E., Sauaia Angela, Kenny-Moynihan Mary, Burch Jon M., Galloway Ben (1997). Predicting Life-threatening Coagulopathy in the Massively Transfused Trauma Patient. The Journal of Trauma: Injury, Infection, and Critical Care.

[CR11] Cullinane DC, Schiller HJ, Zielinski MD, Bilaniuk JW, Collier BR, Como J (2011). Eastern association for the surgery of trauma practice management guidelines for hemorrhage in pelvic fracture-update and systematic review. J Trauma.

[CR12] Demetriades D, Karaiskakis M, Toutouzas K, Alo K, Velmahos G, Chan L (2002). Pelvic fractures: epidemiology and predictors of associated abdominal injuries and outcomes. J Am Coll Surg.

[CR13] Dietrich HH, Dacey RG (2000). Molecular keys to the problems of cerebral vasospasm. Neurosurgery.

[CR14] Eastridge BJ, Starr A, Minei JP, O’Keefe GE, Scalea TM (2002). The importance of fracture pattern in guiding therapeutic decision-making in patients with hemorrhagic shock and pelvic ring disruptions. J Trauma.

[CR15] El-Haj M, Bloom A, Mosheiff R, Liebergall M, Weil YA (2013). Outcome of angiographic embolisation for unstable pelvic ring injuries: factors predicting success. Injury.

[CR16] Ertel W, Keel M, Eid K, Platz A, Trentz O (2001). Control of severe hemorrhage using C-clamp and pelvic packing in multiply injured patients with pelvic ring disruption. J Orthop Trauma.

[CR17] Fang JF, Shih LY, Wong YC, Lin BC, Hsu YP (2009). Repeat transcatheter arterial embolization for the management of pelvic arterial hemorrhage. J Trauma.

[CR18] Fang JF, Shih LY, Wong YC, Lin BC, Hsu YP (2011). Angioembolization and laparotomy for patients with concomitant pelvic arterial hemorrhage and blunt abdominal trauma. Langenbeck's Arch Surg.

[CR19] Fangio Pascal, Asehnoune Karim, Edouard Alain, Smail Nadia, Benhamou Dan (2005). Early Embolization and Vasopressor Administration for Management of Life-Threatening Hemorrhage from Pelvic Fracture. The Journal of Trauma: Injury, Infection, and Critical Care.

[CR20] Frandon J, Arvieux C, Thony F (2016). Indications for embolization in a French level 1 trauma center. J Visc Surg.

[CR21] Frevert S, Dahl B, Lönn L (2008). Update on the roles of angiography and embolisation in pelvic fracture. Injury.

[CR22] Fu CY, Hsieh CH, Wu SC, Chen RJ, Wang YC, Shih CH (2013). Anterior-posterior compression pelvic fracture increases the probability of requirement of bilateral embolization. Am J Emerg Med.

[CR23] Gourlay D, Hoffer E, Routt M, Bulger E (2005). Pelvic angiography for recurrent traumatic pelvic arterial hemorrhage. J Trauma.

[CR24] Graham RNJ (2012). Battlefield radiology. Br J Radiol.

[CR25] Hakim Wasim, Kamanahalli Raghavendra, Dick Elizabeth, Bharwani Nishat, Fetherston Shirley, Kashef Elika (2016). Trauma whole-body MDCT: an assessment of image quality in conventional dual-phase and modified biphasic injection. The British Journal of Radiology.

[CR26] Hauschild O, Aghayev E, Von Heyden J, Strohm PC, Culemann U, Pohlemann T (2012). Angioembolization for pelvic hemorrhage control: results from the German pelvic injury register. J Trauma Acute Care Surg.

[CR27] Ierardi AM, Duka E, Lucchina N, Floridi C, Martino ADE, Donat D et al (2016) EMERGENCY RADIOLOGY SPECIAL FEATURE : REVIEW ARTICLE The role of interventional radiology in abdominopelvic trauma October 2015 10.1259/bjr.20150866 PMC4985465 26642310

[CR28] Ierardi AM, Piacentino F, Fontana F, Petrillo M, Floridi C, Bacuzzi A (2015). The role of endovascular treatment of pelvic fracture bleeding in emergency settings. Eur Radiol.

[CR29] Karadimas EJ, Nicolson T, Kakagia DD, Matthews SJ, Richards PJ, Giannoudis PV (2011). Angiographic embolisation of pelvic ring injuries. Treatment algorithm and review of the literature. Int Orthop.

[CR30] Katsura Morihiro, Yamazaki Shin, Fukuma Shingo, Matsushima Kazuhide, Yamashiro Toshimitsu, Fukuhara Shunichi (2013). Comparison between laparotomy first versus angiographic embolization first in patients with pelvic fracture and hemoperitoneum: a nationwide observational study from the Japan Trauma Data Bank. Scandinavian Journal of Trauma, Resuscitation and Emergency Medicine.

[CR31] Kauvar David S., Lefering Rolf, Wade Charles E. (2006). Impact of Hemorrhage on Trauma Outcome: An Overview of Epidemiology, Clinical Presentations, and Therapeutic Considerations. The Journal of Trauma: Injury, Infection, and Critical Care.

[CR32] Kimbrell BJ, Velmahos GC, Chan LS, Demetriades D (2004). Angiographic embolization for pelvic fractures in older patients. Arch Surg.

[CR33] Lee C, Kaufman JA, Fan CM, Geller SC, Brewster DC, Cambria RP (2000). Clinical outcome of internal iliac artery occlusions during endovascular treatment of aortoiliac aneurysmal diseases. J Vasc Interv Radiol.

[CR34] Lustenberger T, Wutzler S, Störmann P, Laurer H, Marzi I (2015). The role of angio-embolization in the acute treatment concept of severe pelvic ring injuries. Injury.

[CR35] Manson T, O’Toole RV, Whitney A, Duggan B, Sciadini M, Nascone J (2010). Young-burgess classification of pelvic ring fractures: does it predict mortality, transfusion requirements, and non-orthopaedic injuries?. J Orthop Trauma.

[CR36] Matityahu Amir, Marmor Meir, Elson Joshua Knute, Lieber Corey, Rogalski Gregory, Lin Cindy, Belaye Tigist, Miclau Theodore, Kandemir Utku (2013). Acute Complications of Patients With Pelvic Fractures After Pelvic Angiographic Embolization. Clinical Orthopaedics and Related Research.

[CR37] Metsemakers WJ, Vanderschot P, Jennes E, Nijs S, Heye S, Maleux G (2013). Transcatheter embolotherapy after external surgical stabilization is a valuable treatment algorithm for patients with persistent haemorrhage from unstable pelvic fractures: outcomes of a single Centre experience. Injury.

[CR38] Michailidou M, Velmahos GC, Van Der Wilden G, Alam HB, De Moya M, Chang Y (2012). “Blush” on trauma computed tomography: not as bad as we think!. J Trauma Acute Care Surg.

[CR39] Miller PR, Moore PS, Mansell E, Meredith JW, Chang MC (2003). External fixation or arteriogram in bleeding pelvic fracture: initial therapy guided by markers of arterial hemorrhage. J Trauma.

[CR40] National Institute for Health and Care Excellence (2016) Major trauma: assessment and initial management (NICE Guideline 39). Available at: https://www.nice.org.uk/guidance/ng39. Accessed 1 June 2018 26913320

[CR41] Niola R, Pinto A, Sparano A, Ignarra R, Romano L, Maglione F (2012). Arterial bleeding in pelvic trauma: priorities in angiographic embolization. Curr Probl Diagn Radiol.

[CR42] Obaro RO, Sniderman KW (1995). Avascular necrosis of the femoral head as a complication of complex embolization for severe pelvic haemorrhage. Br J Radiol.

[CR43] Ptohis ND, Charalampopoulos G, Abou Ali AN, Avgerinos ED, Mousogianni I, Filippiadis D (2017). Contemporary role of embolization of solid organ and pelvic injuries in Polytrauma patients. Front Surg.

[CR44] Sauaia A, Moore FA, Moore EE, Moser KS, Brennan R, Read RA (1995). Epidemiology of trauma deaths: a reassessment. J Trauma.

[CR45] Schwartz DA, Medina M, Cotton BA, Rahbar E, Wade CE, Cohen AM (2014). Are we delivering two standards of care for pelvic trauma? Availability of angioembolization after hours and on weekends increases time to therapeutic intervention. J Trauma Acute Care Surg.

[CR46] Shapiro M, McDonald AA, Knight D, Johannigman JA, Cuschieri J (2005). The role of repeat angiography in the management of pelvic fractures. J Trauma.

[CR47] Sieber Paul R. (1994). Bladder Necrosis Secondary to Pelvic Artery Embolization: Case Report and Literature Review. The Journal of Urology.

[CR48] Smith W, Williams A, Agudelo J, Shannon M, Morgan S, Stahel P (2007). Early predictors of mortality in hemodynamically unstable pelvis fractures. J Orthop Trauma.

[CR49] Starr AJ, Griffin DR, Reinert CM, Frawley WH, Walker J, Whitlock SN (2002). Pelvic ring disruptions: prediction of associated injuries, transfusion requirement, pelvic arteriography, complications, and mortality. J Orthop Trauma.

[CR50] Stephen DJG, Kreder HJ, Day AC, McKee MD, Schemitsch EH, ElMaraghy A (1999). Early detection of arterial bleeding in acute pelvic trauma. J Trauma.

[CR51] Suzuki T, Shindo M, Kataoka Y, Kobayashi I, Nishimaki H, Yamamoto S (2005). Clinical characteristics of pelvic fracture patients with gluteal necrosis resulting from transcatheter arterial embolization. Arch Orthop Trauma Surg.

[CR52] Tanizaki S, Maeda S, Matano H, Sera M, Nagai H, Ishida H (2014). Time to pelvic embolization for hemodynamically unstable pelvic fractures may affect the survival for delays up to 60 min. Injury.

[CR53] The Trauma Audit & Research Network (TARN 2015) The Injury Severity Score (ISS) & Abbreviated Injury Scale (AIS). Available at: https://www.tarn.ac.uk/Content.aspx?ca=4&c=3117. Accessed 1 June 2018

[CR54] Tran TLN, Brasel KJ, Karmy-Jones R, Rowell S, Schreiber MA, Shatz DV (2016). Western trauma association critical decisions in trauma: management of pelvic fracture with hemodynamic instability - 2016 updates. J Trauma Acute Care Surg.

[CR55] Travis T, Monsky WL, London J, Danielson M, Brock J, Wegelin J (2008). Evaluation of short-term and long-term complications after emergent internal iliac artery embolization in patients with pelvic trauma. J Vasc Interv Radiol.

[CR56] Velmahos GC, Chahwan S, Falabella A, Hanks SE, Demetriades D (2000). Angiographic embolization for intraperitoneal and retroperitoneal injuries. World J Surg.

[CR57] Velmahos GC, Chahwan S, Hanks SE, Murray JA, Berne TV, Asensio J (2000). Angiographic embolization of bilateral internal iliac arteries to control life-threatening hemorrhage after blunt trauma to the pelvis. Am Surg.

[CR58] Velmahos GC, Toutouzas KG, Vassiliu P, Sarkisyan G, Chan LS, Hanks SH (2002). A prospective study on the safety and efficacy of angiographic embolization for pelvic and visceral injuries. J Trauma.

[CR59] White Christopher E., Hsu Joseph R., Holcomb John B. (2009). Haemodynamically unstable pelvic fractures. Injury.

[CR60] Wong YC, Wang LJ, Ng CJ, Tseng IC, See LC (2000). Mortality after successful transcatheter arterial embolization in patients with unstable pelvic fractures: rate of blood transfusion as a predictive factor. J Trauma.

